# Mapping innovations in partograph technologies: a scoping review from 2000 to 2025

**DOI:** 10.3389/fgwh.2025.1618317

**Published:** 2025-12-04

**Authors:** Dereje Bayissa Demissie, Doreen Kainyu Kaura, Kristiaan Schreve

**Affiliations:** 1 Department of Nursing and Midwifery, Faculty of Medicine and Health Sciences, Stellenbosch University, Cape Town, South Africa; 2 School of Nursing, Faculty of Community Health Sciences, University of the Western Cape, Cape Town, South Africa; 3 Department of Mechanical and Mechatronic Engineering, Stellenbosch University, Cape Town, South Africa

**Keywords:** cervix, digital vaginal examination, e-partograph, labour progress, mobile-partograph digital paperless partograph, digital partograph

## Abstract

**Background:**

Digital technologies like the electronic partograph have revolutionised the documentation of progress of labour and birth. The purpose of the electronic partograph is to improve documentation of the progress of the intrapartum period by addressing challenges in partograph use. The tool provides real-time decision support, enhances data entry, and increases access and coordination of information for informed decision-making. Further research is required to map innovations in partograph technologies embedded in data documentation and labour progress monitoring.

**Objective:**

The aim of this scoping review is to map innovations in partograph technologies based on studies published between 2000 and 2025.

**Methods:**

This scoping review followed the five-step framework established by Arksey and O'Malley as well as the population, concepts, and contexts model. A comprehensive search was conducted across seven databases using refined keywords. Data were extracted, charted, synthesised, and summarised.

**Result:**

A total of 13 original articles—studying 8,655 women in labour—were included in this review. The studies evaluated an electronic or digital paperless partograph, assessing its effectiveness and user-friendliness compared with the WHO/modified WHO partograph. This scoping review highlights that digital partographs, especially mobile applications and digital paperless versions, are practical tools for improving labour monitoring globally.

**Conclusion:**

This scoping review found that digital paperless and novel partograph designs show promise for improving labour monitoring, particularly in resource-limited settings. The adoption of these tools can streamline documentation, enhance communication among healthcare providers, and facilitate timely interventions. This review recommends integrating ultrasound-based digital tools into labour monitoring for improved diagnostic accuracy and patient comfort.

**Systematic Review Registration:**

https://osf.io/m96tw/

## Introduction

Despite the recommendation of the WHO on the use of the partograph for tracking labour progress and identifying complications, its utilisation in many low- and middle-income countries (LMICs) remains suboptimal, ranging between 20% and 80% (1). Several barriers contribute to this limited uptake, including inadequate training, insufficient knowledge and resources among healthcare providers, poor attitudes and clinical skills, and systemic challenges such as weak clinical leadership and lack of quality assurance mechanisms. Strategies to improve adherence include on-the-job training, supportive supervision, and addressing context-specific barriers. While emerging technologies may help overcome these challenges, further evaluation is required to determine their feasibility and effectiveness (1, 2).

Digital technologies such as the electronic partograph are transforming how labour and birth are documented. Recent studies have shown that the digital paperless partograph is more user-friendly and preferred by healthcare providers, resulting in higher completion rates and improved staff compliance (3). It has demonstrated effectiveness comparable to the WHO partograph in managing labour, with similar maternal and perinatal outcomes (4). In high-volume settings, the digital paperless partograph is simpler to use and may serve as a viable alternative to the WHO-modified version (5). Overall, it is an efficient, user-friendly tool for monitoring labour and promoting safe delivery practices (3–5).

The systematic review of partograph adherence trends in healthcare services recommends on-the-job training, routine monitoring, and supportive supervision to improve compliance. It emphasises the importance of partograph use in improving maternal and neonatal outcomes and calls for further research to identify effective interventions (1). The developed device digitises the traditional paper partograph, enables simultaneous patient recording, and provides a decision support system to detect abnormalities during labour. The objective of the digital paperless version is to enhance the safety, efficiency, and quality of labour monitoring (6). Future studies should focus on developing effective, non-invasive methods for assessing labour progress while simultaneously collecting data on an electronic “partograph” for accurate documentation with emphasis on the preferences and views of the women in labour (7). The electronic partograph is a tool designed to enhance the care of women in labour by addressing challenges in the use of traditional partographs. It offers real-time decision support, improved data entry, and increased access and transfer of information for effective labour management (8).

Innovations in partograph technologies are increasingly being explored to address existing gaps. This scoping review maps the range of existing innovations in partograph tools and technologies, with a focus on their application in documenting labour progress and birth outcomes. It aims to identify trends, gaps, and opportunities for future research and implementation. This scoping review was conducted on the basis of the population, concept, and context (PCC) framework. See details in Table 1. The population constituted adult women in labour; the concept involved mapping existing innovations in partograph technologies for monitoring progress of labour; and the context was global.

The review aims to answer the following question:

1. What are the existing innovations in partograph technologies used to document the progress of labour and birth globally?

## Methods

This scoping review was carried out in accordance with the method suggested by Arksey and O'Malley (9, 10). The review followed five steps: identifying the research question; searching for relevant studies; selecting appropriate studies; charting the data; and collating, summarising, and reporting results. Recommendations by Levac and colleagues were taken into consideration throughout the process (11). A scoping study approach enables a systematic search, selection, and examination of the literature, as well as synthesis and mapping of evidence to address research questions (9, 10). The Preferred Reporting Items for Systematic Reviews extension for Scoping Reviews (PRISMA-ScR) was used to present the scoping review (11). The PRISMA-ScR provides a reporting guideline comprising 20 essential items and two optional items for this scoping review (11). This guideline facilitates methodological transparency and acceptance of research findings (11). The protocol for this scoping review has been registered with the Open Science Framework (https://osf.io/m96tw/).

Step 1: identifying the research question: the present scoping review aims to answer the following question
1. What existing innovations in partograph technologies have been developed to document the progress of labour and birth globally?
Step 2: identifying relevant studies

The search strategy aimed to locate both published and unpublished studies. Five databases were systematically searched, along with grey literature, and data were extracted, charted, synthesised, and summarised. The search strategy began with a brief initial search of Medline and CINAHL, followed by the use of keywords from the titles and abstracts of articles found in Cochrane Review Library, PubMed, CINAHL, Medline, EMBASE, Scopus, and Web of Science. Grey literature searches were included to formulate research questions, identify and select studies, chart data, and summarise results, using text words and index terms for a comprehensive search strategy (see Table 2). A complete search strategy employing both medical subject headings (MeSH) and keywords was used. Boolean terms (AND and OR) were used to separate keywords, and Medical Subject Headings (MeSH) terms were used in the advanced search of articles. The search strategy, including all identified keywords and index terms, was adapted for each database and/or information source. A snowball approach was used to screen reference lists for additional studies, including secondary searches of relevant articles and systematic reviews. Only English-language studies published between 1 January 2000 and 14 February 2025 were included. The details of the search strategy are presented in Table 2.

**Table 1:** PCC (people, concept, and context) framework used to identify review questions.

People	The population included in this review comprised women of reproductive age (15–49 years), specifically those who were pregnant, in labour, or undergoing delivery
Concept	Electronic partograph, mobile partograph, WHO partograph; Monitoring Techniques (Electronic, Novel, Digital paperless), Labour Progress, Follow-Up
Context	Globally, all available evidence was mapped. • Published since 1 January 2000 to 15 February 2025. • Written in English • All primary studies (quantitative, qualitative, and mixed-method published articles) ○ Quantitative (cross-sectional/ observational), ○ Qualitative (phenomenology, case study) and ○ Mixed-method published articles

**Table 2:** The sample search strategy used to identify relevant articles using keywords.

S.no	Search strategy	Database used	Number of articles
	devices for Measuring) OR examination*) AND digital vaginal) OR digital) AND vaginal) OR vaginal examination) OR Artificial Assisted intelligence) OR digital paperless partograph OR Electronic) OR Follow-Up) OR Mobile-Application) OR Mobile-partograph) OR Partograph-Device) OR active labour) OR labour) OR cervix) OR partograph) OR partograph) or e-partograph or electronic-Touch OR effacement) OR mLabour) OR Monitoring Techniques (Electronic/Novel/Digital paperless), Labour Progress, Follow-Up	PubMed	772

### Study selection

#### Selecting eligible studies

The eligibility criteria of the study were based on the PCC framework established by the Joanna Briggs Institute (9, 11). The study types included primary studies published in peer-reviewed journals, accessible online and through inter-library requests. Non-English language articles were excluded. Eligible articles were uploaded into the EndNote 21 reference management software (12), and duplicates were removed. Title and abstract screening and full-text reviews were conducted independently by two researchers (DD and DK), with a third reviewer weighing in if significant discrepancies could not be resolved.

Step 3: study selection

Two review authors independently evaluated the titles and abstracts of the included articles. Full-text articles were obtained for all titles and abstracts that met the eligibility criteria. The two authors evaluated the full-text publications independently to determine which studies should be included in the review based on eligibility criteria.

### Eligibility criteria

This scoping review included articles that met the following eligibility criteria:

• studies involving pregnant women undergoing labour, delivery follow-up, or labour progress;
• studies that reported evidence of digital partograph technologies used to document vaginal examination to monitor progress of labour;
• studies from any part of the world;
• English-language publications.

#### Scoping review exclusion criteria

The review team excluded articles published in languages other than English because of constraints related to time, funding, and the lack of necessary language skills and resources to handle non-English studies and databases.

Studies that did not focus on digitalised partographs compared with the routine traditional WHO partograph were also excluded.

### Study/source of evidence selection

**Figure 1:**
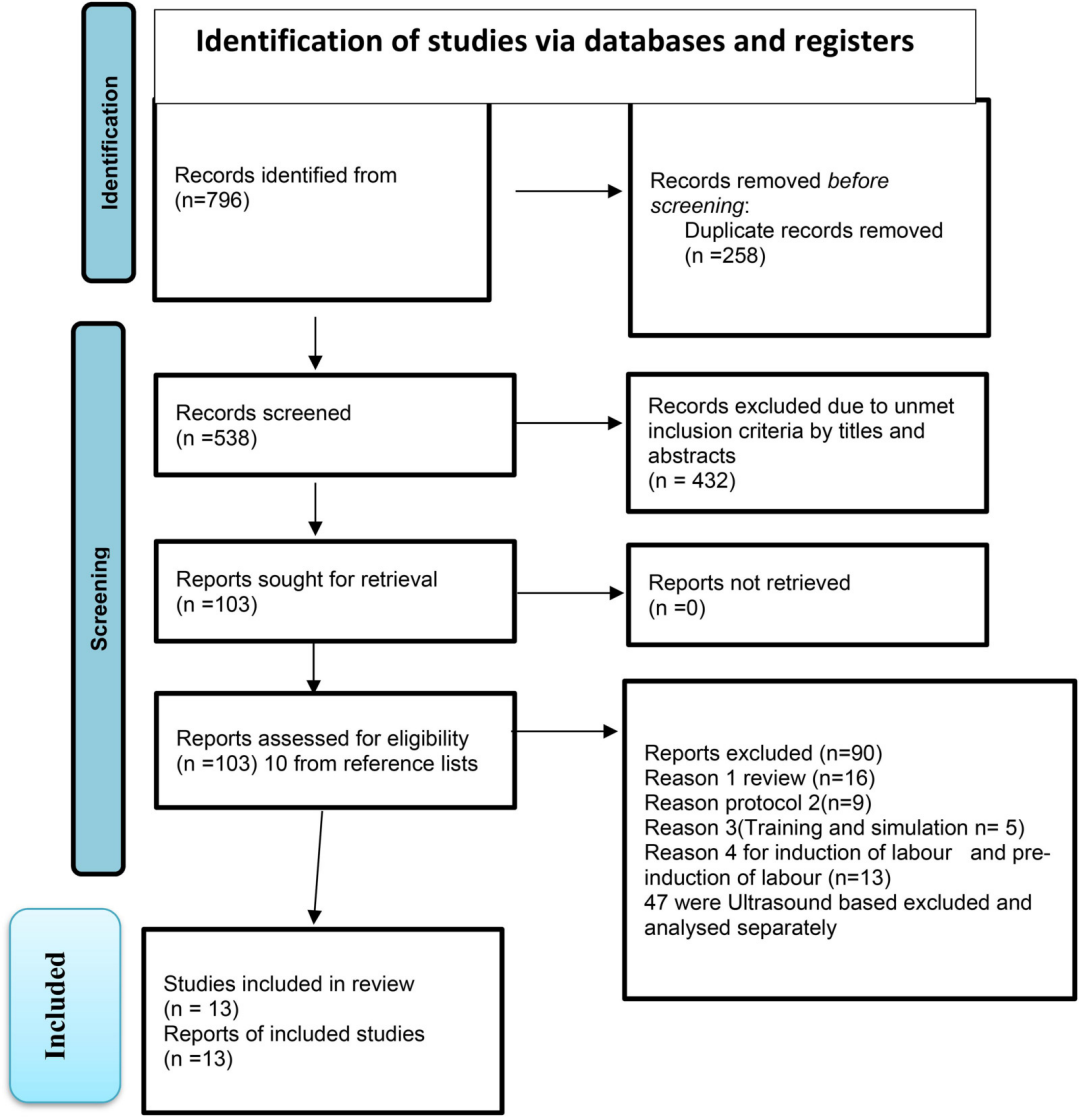
PRISMA-ScR 2020 flow diagram for new systematic reviews, which included searches of databases, registers, and other sources (13).

The two review authors conducted a comprehensive title screening using electronic databases in accordance with the eligibility criteria. All relevant articles were imported into the EndNote library software to remove duplicates and were shared with the review team for the next stage of the study screening and selection process. Duplicate records were checked and removed. A screening process for the abstract and full-text screening phases was developed using the eligibility criteria. The two review authors independently completed the abstract and full-text screening and collected data to include categories. The issue of discrepancies in abstract screening were addressed through discussion among the review team until a consensus was reached. The PRISMA-ScR flow diagram was used to report the screening results (13) (Figure 1).

Step 4: charting the data

Two authors (DD and DK) extracted data from the included articles, refining the data extraction tool with 10 studies. They collected data on study characteristics, including authors, title, country, aim, sample size, design, population, methods, findings, limitations, and partograph technologies related to electronic labour progress, mobile partographs, and monitoring techniques. Data were categorised by the two independent reviewers in a spreadsheet of key factors for easy analysis.

Step 5: collating, summarising, and reporting the results

According to Arksey and O'Malley (10), scoping reviews require thematic frameworks to present narrative accounts of the selected literature. The data were grouped thematically by reading and rereading the data items and grouping similar/related items into iteratively developed themes. A PRISMA-ScR flow diagram was used to demonstrate the literature study selection process and search results (9, 11, 13). A descriptive numerical summary was prepared to present the characteristics of the included studies. A qualitative thematic synthesis was also conducted using a data-driven bottom-up approach. The results were classified under main conceptual categories such as “digitalised vaginal examination, Mobile partograph, and ultrasonic partograph” and “Artificial Intelligence (AI)-assisted vaginal examination, non-intrusive vaginal examination.” Under each category, further data were provided on the characteristics of the article, including but not limited to the total number of studies, study design types, source of data, year of publications, and key findings (see Table 3). Tables and figures were used to present the results, in line with the objectives of the review. The implications of the results were elaborated with consideration given to research and practice to help identify gaps in labour follow-up. The findings will aid in developing a non-intrusive AI-enabled prototype for vaginal examinations and monitoring progress of labour, integrating AI algorithms to analyse and interpret data, ensuring accuracy, and minimising patient discomfort.

**Table 3:** Partograph-based technologies (electronic/novel/digital paperless) used in labour follow-up and monitoring characterised by study setting, sample size, study design, and types of partograph used (2000–2025).

Study's title	Author and year of publication	Study setting/country	Sample size	Study design	Study participants	Technology/application/devices used
Feasibility and effectiveness of electronic vs. Paper partograph on improving birth outcomes	Rahman et al. (4)	Bangladesh	2,918	Prospective crossover study	Women attending deliveries	E-partograph
Evaluation of Digital paperless Partograph as a Bedside Tool in the Management of Labor	Agarwal et al. (14)	India	90	Prospective, observational cohort study	Labouring women	Digital paperless partograph
Study to compare between digital paperless partograph and modified WHO partograph in management of labour	Veena and Anagondanahalli (22)	India	200	Prospective observational study	Women in labour	Digital paperless partograph, WHO partograph
Utility of digital paperless partograph in labour management	Tarannum and Akhtar (2)	India	400	Prospective analytical study	Pregnant women labour	Digital paperless partograph
A comparative study of digital paperless partograph and modified WHO partograph in management of labour	Rohini (20)	India	1,040	Comparative cross-sectional study	Women who were admitted in labour	Digital paperless partograph
WHO labour care guide versus digital paperless partograph for effective management of labour in a tertiary care centre	Ranjan et al. (19)	India	160	Prospective interventional study	Women in labour	Digital paperless partograph
Partograph versus no partograph: effect on labour progress and delivery outcome: a comparative study	Ahmed et al. (15)	India	400	Prospective randomised comparative study	Women in labour	Partograph and no partograph
Comparative study between WHO modified partograph and the digital paperless partograph in management of labour	Metawia et al. (18)	Egypt	300	Prospectively	Women in labour	Digital paperless partograph
DAKSH: digital partograph and intrapartum monitoring mobile application	Megha et al. (17)	India	463	Cross-sectional study	Pregnant + Labour	Digital Partograph and Mobile Application
The digital paperless partograph: can it be effective to replace the WHO modified partograph	Deka et al. (3)	India	400	Hospital-based prospective analytical study	Labouring pregnant	Digital paperless partograph vs WHO partograph
Increasing adherence to plotting e- partograph: a quality improvement project in a rural maternity hospital in India	Jain et al. (16)	India	Not explicitly stated	Quality improvement (QI) project, Plan-Do-Study-Act (PDSA) cycles	Labouring pregnant	E- partograph
Development of a low-intensity light imaging probe for childbirth cervical dilation image acquisition	Takpor et al. (24)	Nigeria	2,880	Design is a proof-of-concept, laboratory	Women in labour	Prototype of a novel low-intensity light imaging probe
Effectiveness of an electronic partograph: a mixed-method, quasi-experimental study among skilled birth attendants in Kenya	Sanghvi et al. (21)	Kenya	1,884	Quasi-Experimental Study	Active labour	Android tablet application (e-partograph)

## Result

### Study inclusion

The full text of selected studies was analysed in detail against the inclusion criteria by two independent reviewers. The exclusion criteria, at the full-text stage, were documented and included in the scoping review. Disagreements between reviewers were resolved through discussion or consultation with a third reviewer. The study selection process was reported in full and illustrated using a PRISMA-ScR flow diagram.

A total of 796 articles were screened by the two reviewers, with disagreements resolved in four cases. Of these, 151 articles underwent full-text review, resulting in the retrieval of 103 articles, of which 13 met the inclusion criteria for the scoping review (2–5, 14–22). The number of studies identified, screened, included, and excluded at each stage is provided in the PRISMA flow chart (Figure 1). The primary reasons for exclusion at the full-text stage were ineligible outcomes and interventions.

### Study characteristics

A total of 13 original articles were included in this scoping review (2–5, 14–22), where the studies were conducted across a variety of geographical locations, including Europe (UK, France, Norway, and Italy,), Asia (India, China, and South Korea), Africa (Ghana, Kenya, Nigeria, and Egypt), South America (Brazil), the Middle East (Israel, Turkey, and Iran), and North America (USA). This provides a broad perspective on labour management practices across different healthcare systems.

The studies involved a diverse group of participants in labour, including those with low-risk and singleton pregnancies. The sample sizes varied considerably, ranging from small pilot studies with approximately 25 participants in Belgium (23) to larger cohort studies with several hundred women [up to 2,880 in some cases in Nigeria (24)].

Most of the studies employed prospective observational cohort designs, which allowed for the systematic collection of data on labour progress using both traditional methods (digital vaginal examinations, partographs) and newer techniques (ultrasound, AI-assisted tools).

As indicated in Supplementary Table S1, the research landscape on vaginal examination and labour follow-up is characterised by studies conducted across diverse settings, participants, and study designs, reflecting the growing interest in improving labour management practices worldwide. Supplementary Table S1 provides a concise overview of the research landscape in terms of digital devices and technologies for labour monitoring.

### Partograph-based technologies (electronic, novel, and digital paperless) used in labour follow-up

Partograph-based approaches were reported in 13 studies involving 8,255 women. These studies documented labour monitoring using a range of partograph techniques, from the traditional format to the modified WHO partograph, in both electronic and digital paperless forms. The studies were primarily conducted in India, Bangladesh, Egypt, and Kenya, focusing on tertiary care centres and rural maternity hospitals. The sample sizes ranged from 90 to 2,918 participants. The technologies analysed included the e-partograph, digital paperless partograph, WHO partograph, Digital Partograph Mobile Application (DAKSH), and Android tablet-based e-partograph (2–5, 14–22). This review highlights the use of digital partographs in vaginal examinations and labour follow-ups. The included studies assessed the effectiveness and user-friendliness of the digital partograph compared with the WHO and modified WHO partograph. The adoption of the digital partograph—particularly mobile applications, digital paperless partographs, and mobile applications—offers practical solutions for improving labour monitoring globally, including the potential to improve labour management in low-resource settings. Digital paperless partographs are especially user-friendly and effective in such settings (14, 22). Mobile applications such as DAKSH enhance accessibility in primary healthcare centres. Tablet-based e-partograph systems integrate decision support features for skilled birth attendants (17, 21). These technologies are widely used to improve labour management and efficiency. Further details are provided in Table 3.

### Innovations in partograph technologies

Between 2000 and 2025, partograph technologies have evolved from paper-based charts to digital, application-based tools accessible via mobile devices. These innovations support nurses and midwives in real-time decision-making during labour. Key features include simplified data entry, enhanced visualisation, automated alerts, and integrated referral systems. Digital partographs improve documentation accuracy, support protocol adherence, and facilitate recognition of complications, while enabling secure data storage and retrospective analysis. Despite challenges like poor infrastructure and lack of training, innovations in partographs offer scalable solutions that enhance clinical efficiency and promote equitable maternal care across diverse healthcare settings (2–5, 14–22).

### Key findings on partograph-based technologies (electronic, novel, and digital paperless) for labour follow-up and progress monitoring

The scoping review of electronic partograph (e-partograph) studies found them to be feasible and effective in improving user rates, reducing caesarean births and prolonged labour in Bangladesh, and enhancing the quality of care in rural Indian maternity hospitals (4, 16). In Kenya, an Android tablet-based e-partograph led to significant improvements in foetal outcomes and increased use of interventions to maintain normal labour, which providers regarded as adoptable (21). These findings highlight the potential of the e-partograph in improving maternal and neonatal outcomes.

Studies from India and Egypt revealed the digital paperless partograph to be efficient, user-friendly, and time-saving. It performed comparably to the WHO partograph in labour management and was noted to be well suited for high-volume settings with limited staff and resources. The digital paperless partograph can be easily utilised at peripheral health centres, potentially reducing maternal mortality without additional costs (2, 3, 14, 18, 20). Previous studies have evaluated the Digital Partograph Mobile Application (DAKSH), indicating its potential to improve patient care and reduce healthcare work burdens, with improved patient records maintained through multiple checks within the application (17). Another study of labour monitoring using partograph compared with no partograph reported that the use of a partograph is associated with better monitoring of labour progress and improved delivery outcomes (15). Further details are provided in Table 4.

**Table 4:** Key findings on partograph-based technologies (electronic/novel/digital paperless) used in labour follow-up and monitoring the progress of labour, 2000–2025.

Author and year	Aims and objectives	Conclusion and recommendations
Rahman et al. (4)	Test the feasibility and effectiveness of implementing an e-partograph, for the first time, in 2 public hospitals in Bangladesh.	The e-partograph significantly improved usage rates and was linked to reduced caesarean births and prolonged labour. It supported timely obstetric decisions and is recommended for inclusion in labour management guidelines. Scaling its use in public and private hospitals across Bangladesh could enhance maternal and neonatal care quality while reducing unnecessary interventions and complications such as birth asphyxia.
Agarwal et al. (14)	To evaluate prospectively the use of a digital paperless partograph as a bedside tool in the management of labour.	The study found that the digital paperless partograph was convenient and effective in managing labour, with a mean delivery duration of 4.3 h after Alert ETD.
		The study's conclusion is that the digital paperless partograph is similar to the World Health Organization's recommendation for partograph, with a 4-h action line for intervention for prolonged labour.
		The electronic version of the partograph is a state-of-the-art application that could be accessed through a smart phone or tablet pc or computer device.
Veena and Anagondanahalli (22)	To know the efficacy and user-friendliness of a digital paperless partograph in comparison with a WHO partograph in the monitoring and management of labour.	Debdas' digital paperless partograph is a simple, safe, and cost-effective tool for labour monitoring. It outperformed the WHO partograph in documentation, usability, and efficiency, making it ideal for high-volume, low-resource settings. Preferred by staff, it enables timely referrals and improved outcomes. Further training is recommended to reduce maternal and neonatal mortality and enhance community-level care.
Tarannum and Akhtar (2)	To evaluate the effectiveness of the digital paperless partograph as a bedside tool and its comparison with a WHO-modified partograph.	The digital paperless partograph demonstrated efficiency comparable to the WHO-modified partograph, with a mean delivery time of 3.57 h. Its simplicity and reduced time demands make it suitable for resource-limited settings like India. Given its usability and effectiveness, it presents a viable alternative for managing labour in high-volume environments with limited healthcare personnel.
Rohini (20)	To compare the digital paperless partograph and the modified WHO partograph in the management of labour.	The digital paperless partograph, a simple and graph-less 20-s tool, is as effective as the WHO partograph in detecting abnormal labour and improving outcomes. It is ideal for low-resource, high-volume settings and can be implemented at peripheral health centres without added cost. Its ease of use supports timely reassessment and may help reduce maternal mortality.
Ranjan et al. (19)	To compare the WHO Labour Care Guide and the digital paperless partograph for effective management of labour in a tertiary care centre.	The digital paperless partograph acts as an efficient tool in labour monitoring in a setup with high patient load and poor manpower as it needs minimum training and skill when compared with WHO Labour Care Guidelines. Both are equally effective in preventing prolonged labour and other labour-related complications.
		The study found that the digital paperless partograph was as effective as the WHO partograph in monitoring labour and determining further management, as both partographs help prevent prolonged and obstructed labour. The digital paperless partograph acts as an efficient tool in labour monitoring in a setup with high patient load and less manpower as it needs minimum training and skill when compared with WHO Labour Care Guidelines.
Ahmed et al. (15)	To compare the effect on labour progress and delivery outcome between the partograph and no partograph.	The partograph is a graphical representation of major events in active labour, allowing for easy viewing of foetal and maternal parameters. It helps manage labour, reduces active phases, and reduces the need for augmentation. It also helps in assessing labour progress, maternal and foetal parameters, and deciding when an operative intervention is needed, ultimately resulting in a healthy mother and baby. The use of the partograph, when compared with no partograph plotting in active labour, is associated with better monitoring of labour progress as well as delivery outcome in the form of a healthy mother and a healthy child.
Metawia et al. (18)	To assess the user-friendliness and effectiveness of the digital paperless partograph in the management of labour.	The study found that the digital paperless partograph was as efficient as the WHO partograph in monitoring labour and deciding management, as it aids in the prevention of prolonged and obstructed labour. A digital paperless partograph is recommended in high patient load and limited staff settings as it is simpler to use and requires less time for application.
		Increased training and greater knowledge dissemination about partograph protocols are also recommended.
Megha et al. (17)	To evaluate the feasibility and acceptability of a mobile partograph in low-resource primary healthcare centres.	The DAKSH application integrates a digital partograph with features like complication alerts, referral systems, antenatal history, and laboratory investigations to support labour monitoring from admission to discharge. Developed through scenario testing and user feedback, it improves documentation and reduces workload. Preliminary data suggest enhanced patient care, although further research is needed. The study highlights strengths in daily data monitoring and evaluation, while noting limitations in data uniformity and reliability across hospital settings.
Deka et al. (3)	To determine whether the digital paperless partograph can replace the WHO partograph to monitor labour and aid in decision-making.	The study found that the digital paperless partograph was as efficient as the WHO partograph in monitoring labour and deciding on further management.
		However, the digital paperless partograph was easier to maintain and more user-friendly and hence could be easily plotted even by those with minimal formal training on its use.
		Thus, it can serve to replace the WHO partograph, particularly in areas with high patient volume and shortage of manpower.
Jain et al. (16)	To improve the rates of e-partograph plotting in all eligible women in the labour room from the existing 30% to 90% in 6 months through a quality improvement (QI) process.	The e-partograph quality improvement project implemented in a rural maternity hospital in India has led to a sustained decrease in obstructed and prolonged labour and associated complications, as well as a decrease in neonatal admissions for birth asphyxia.
		The project suggests that a QI approach can improve adherence to e-partograph plotting, resulting in improved maternal health services in rural maternity hospitals.
Sanghvi et al. (21)	To assess the effectiveness of an Android tablet-based electronic, labour clinical decision support application (e-Partograph) in limited-resource settings.	Use of the e-partograph resulted in significant improvements in foetal outcomes and use of interventions to maintain normal labour compared with the paper partograph. SBAs using the partograph were also at least as, and often more, compliant in recording measurements during labour compared with SBAs using the paper partograph. They noted that the alerts prompted them to take timely clinical action. As the WHO developed a revised partograph that reflected its 2018 intrapartum care recommendations, the e-partograph has great potential to improve the quality of care and outcomes through consistent and meaningful labour monitoring and clinical care.
		The use of the e-partograph was associated with improvements in adherence to recommendations for routine labour care and a reduction in adverse foetal outcomes, with providers reporting adoptability without undue effort.
		Continued development of the e-partograph, including incorporating new clinical rules from the 2018 WHO recommendations on intrapartum care, will improve labour monitoring and quality care at all levels of the health system.

## Discussion

This scoping review reveals that electronic partographs (e-partographs) are increasingly recognised as feasible and effective tools for improving labour monitoring. Evidence from Bangladesh and rural India shows that e-partographs enhance user compliance, reduce caesarean section rates, and shorten prolonged labour durations, contributing to better quality of maternal care (2, 16). Consistent with this result, a study in Tanzania demonstrated rapid skill acquisition among skilled birth attendants (SBAs) using the e-partograph. Across 84 shifts monitoring 103 women, 87%–91% of SBAs completed tasks confidently by their first shift, reaching full proficiency by the fifth shift. Most SBAs (93%) reported comfort and efficiency in using the tool, highlighting its usability and adaptability in clinical settings (8). Further evidence from Ethiopia reinforces these findings, where two-thirds of obstetric healthcare providers expressed intent to adopt mobile-based partographs. Their motivation was strongly influenced by perceived usefulness, ease of use, relevance to their roles, and positive attitudes towards digital tools (25). Together, these studies suggest that e-partographs are not only technically viable but also well accepted by frontline healthcare workers. Their integration into labour management protocols could significantly improve decision-making, reduce complications, and enhance maternal and neonatal outcomes, particularly in low-resource settings.

This review underscores the growing potential of mobile-based partograph technologies in improving labour monitoring and maternal outcomes. A study from Kenya demonstrated that an Android tablet-based e-partograph significantly enhanced foetal outcomes and supported timely interventions to maintain normal labour. Healthcare providers found the tool to be adoptable and practical in clinical settings (20). These findings align with those of earlier research suggesting that digital paperless partographs can effectively replace the WHO-modified partograph, offering a more efficient and user-friendly alternative for labour monitoring and safe delivery (3–5). The consistent usability and acceptability across different contexts highlight the importance of designing mobile-based partographs that meet both job requirements and user expectations. Such tools not only streamline clinical workflows but also empower frontline staff to make timely decisions, particularly in resource-constrained environments. Their successful adoption depends on intuitive design, relevance to clinical tasks, and integration into existing maternal health systems. This review found that digital paperless partographs, as evaluated across studies from India and Egypt, were as effective as the WHO-modified partograph in monitoring labour and guiding clinical decision-making. These tools offer a simpler, time-efficient, and user-friendly alternative, making them particularly suitable for settings with high patient volumes and limited staffing (1, 5, 14, 18, 19). Importantly, the digital paperless partograph demonstrated delivery durations comparable to those managed with the WHO partograph, indicating clinical reliability. Its ease of use and minimal training requirements make it highly adaptable for peripheral health centres, where resources are often constrained. Moreover, its implementation does not incur additional costs, making it a practical solution for improving maternal care and potentially reducing maternal mortality in low-resource environments (3–5). These findings suggest that the digital paperless partograph could serve as a scalable and sustainable innovation in labour monitoring, especially in underserved healthcare systems.

This scoping review identified the potential of DAKSH to enhance patient care and reduce the workload of healthcare providers. Developed through iterative testing and user feedback, DAKSH includes built-in checks that improve the completeness and accuracy of patient records, supporting better clinical documentation and decision-making (17). The value of partograph use is further reinforced by a study conducted by Ahmed et al., which showed that labour monitored with a partograph, as compared to no partograph, resulted in improved tracking of labour progress and better delivery outcomes (15). These findings emphasise the importance of structured monitoring tools in improving maternal and neonatal care. In addition, previous studies have shown that tablet-based electronic partographs address common challenges in traditional partograph use. They offer real-time decision support, streamline data entry, and improve access to clinical information, all of which contribute to more effective labour management (8). These digital tools not only enhance the quality of care but also support timely interventions, coordination among providers, and better health outcomes. Collectively, the evidence suggests that mobile- and tablet-based partograph applications, when well designed and integrated into routine practice, can significantly strengthen intrapartum care, especially in resource-constrained settings.

### Strengths and limitations of the study

This scoping review employed a rigorous search and screening strategy to ensure comprehensiveness and minimise bias. It adhered to a well-established methodological framework and incorporated a relevant tool to assess the risk of bias. However, the review was limited to publications in the English language, which may have inadvertently excluded valuable insights from studies published in other languages.

While the review underscores the potential of digital partographs to enhance labour monitoring and maternal outcomes, several implementation challenges must be addressed to support their effective adoption in LMICs.

Infrastructure limitations remain a major barrier. Many rural and under-resourced health facilities lack reliable electricity, internet connectivity, and access to digital devices—basic prerequisites for operating electronic partograph systems. Without these foundational elements, even the most intuitive applications cannot be effectively deployed or sustained.

Digital literacy among healthcare workers is another critical concern. Although evidence suggests that digital partographs are generally well received, their successful use depends on the ability of frontline staff to navigate and interpret digital tools. In many LMICs, nurses and midwives may have limited exposure to digital health technologies, necessitating targeted training and ongoing support to build confidence and competence.

Sustainability of technology adoption also presents a significant challenge. While pilot projects often benefit from donor funding, technical assistance, and close oversight, scaling these tools across national health systems requires long-term investment, integration into existing workflows, and alignment with national health policies. Without clear strategies for system maintenance, software updates, and user support, digital partograph systems risk becoming underutilised or abandoned. Moreover, data privacy and security must be carefully considered, particularly when patient information is stored or transmitted electronically. Ensuring compliance with ethical standards and local regulations is essential to protect patient rights and foster trust in digital health solutions.

## Conclusion and recommendation

In summary, the current research landscape demonstrates a clear shift towards more objective, technology-driven approaches to labour monitoring. Digital partographs—particularly those delivered via mobile- and tablet-based applications—show strong potential to enhance clinical decision-making, reduce adverse outcomes, and improve the overall quality of maternal care.

However, while these technologies offer significant clinical advantages, their successful implementation in LMICs demands more than technical innovation alone. Key challenges include infrastructural constraints such as unreliable electricity and internet connectivity, which can impede the consistent use of digital tools in rural or under-resourced settings. Furthermore, limited digital literacy among healthcare workers remains a barrier, as many frontline providers may lack the training or confidence to use new technologies effectively without ongoing support.

Sustainability is another critical concern. Many digital health interventions are introduced through externally funded pilot projects, yet long-term adoption hinges on integration into national health systems, alignment with policy frameworks, and sustained investment in maintenance and capacity-building. Without these elements, there is a risk of abandonment or underutilisation.

This review also underscores the importance of designing user-centred and context-appropriate tools that align with the workflows and expectations of healthcare providers. When effectively integrated, digital partographs can support real-time decision-making, improve data quality, and facilitate timely referrals—ultimately contributing to better maternal and neonatal outcomes.

In conclusion, digital partographs represent a promising innovation in maternal and neonatal healthcare. To realise their full potential, coordinated efforts among practitioners, policymakers, and researchers will be essential to overcome implementation barriers and ensure equitable access across diverse healthcare settings.

## Data Availability

The original contributions presented in the study are included in the article/Supplementary Material; further inquiries can be directed to the corresponding author.
